# Drug Induced Bullous Lesion Caused By Valproic Acid in Bipolar Affective Disorder:A Case Report

**DOI:** 10.1192/j.eurpsy.2023.2139

**Published:** 2023-07-19

**Authors:** H. D. Polat, D. Göverti

**Affiliations:** 1 ERENKOY MENTAL AND NERVOUS DISEASES TRAINING AND RESEARCH HOSPITAL, İstanbul, Türkiye

## Abstract

**Introduction:**

Bipolar Affective Disorder(BAPD) is characterized by variations in mood from elation and/or irritability to depression.Valproic acid(VPA) is indicated for the treatment of acute manic episodes in BPAD.The use of VPA can be limited by either loss or lack of efficacy or by adverse drug reactions. Stevens-Johnson syndrome(SJS) toxic epidermal necrolysis(TEN) are the rare but fatal cutaneous adverse drug reactions for VPA.

**Objectives:**

We wanted to draw attention that drug induced bullous lesions which has been seen rarely in the literature caused by valproic acid in bipolar disorder.

**Methods:**

We examined the side effects of valproic acid in one of our patients with bipolar affective disorder using our observations and laboratory tests.

**Results:**

A 41 year old man was admitted to the our hospital with complaints of decreased amount of sleep, increased amount of speech, skepticism,irritability and dysphoric mood.The patient who was followed up with a diagnosis of BPAD for about 10 months,attempted suicide by taking lithium 2 days before his hospitalization.Considering that it was a mixed episode and the prophylactic effect of lithium was insufficient,VPA 1000 mg/day and Risperidone 2 mg/day treatment were started.Risperidone was increased to 4 mg/day because psychotic symptoms persisted.Valproic acid dose was increased to 1000 to 1500mg/day after the Valproic acid blood level reached 55.28 in the follow-ups.After 5 days 2 bullous lesions developed on the lower extremity of the patient.Routine laboratory investigations were within normal limits.When we consult the patient with the dermatologist;the dermatologist recommended that the lesion be fixed drug eruption and that valproic acid should be discontinued if possible.It was thought that the lesions of the patient who did not have dermatological disorders and did not describe insect bites,might be due to valproic acid. In addition to all these,the patient’s mother had pemphigus vulgaris.The patient’s valproic acid drug was discontinued and lithium was started.Risperidone treatment was continued.In the follow-ups,the patient’s bullous lesions regressed and no new lesion formation was observed.

**Image:**

**Figure:**
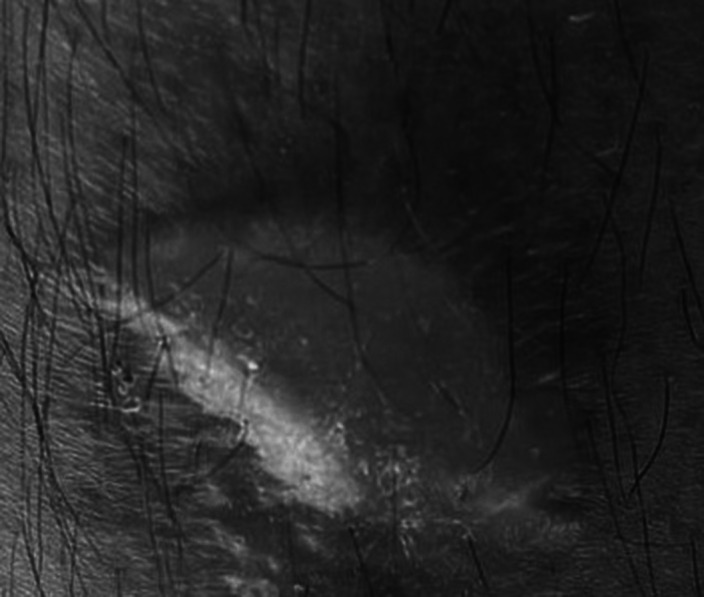

**Conclusions:**

The differential diagnosis of bullous lesions at first may appear overwhelming.In this case traumatic bulla,Pemphigus vulgaris,drug induced bulla,Fixed drug eruption,Steven Johnson Syndrome were among our prediagnosis.Cutaneous drug eruptions associated with VPA can range from maculopapular eruption to severe Stevens-Johnson syndrome or toxic epidermal necrolysis.We were worried that the patient had SJS, but it remained only bullous lesions.We could not biopsy the patient lesions to understand the underlying cause but development of bullous lesions with the initiation of valproate and subsequent remission of the lesions with the discontinuation of the drug and subsequent course clearly suggests a causal relation between valproate and skin lesions.

**Disclosure of Interest:**

None Declared

